# Supplementary figures and images for: Characterization of a novel compound that promotes myogenesis via Akt and transcriptional co-activator with PDZ-binding motif (TAZ) in mouse C2C12 cells
Source: PLoS One. 2020 Apr 8;15(4):e0231265. doi: 10.1371/journal.pone.0231265 (PMC7141682; doi:10.1371/journal.pone.0231265)

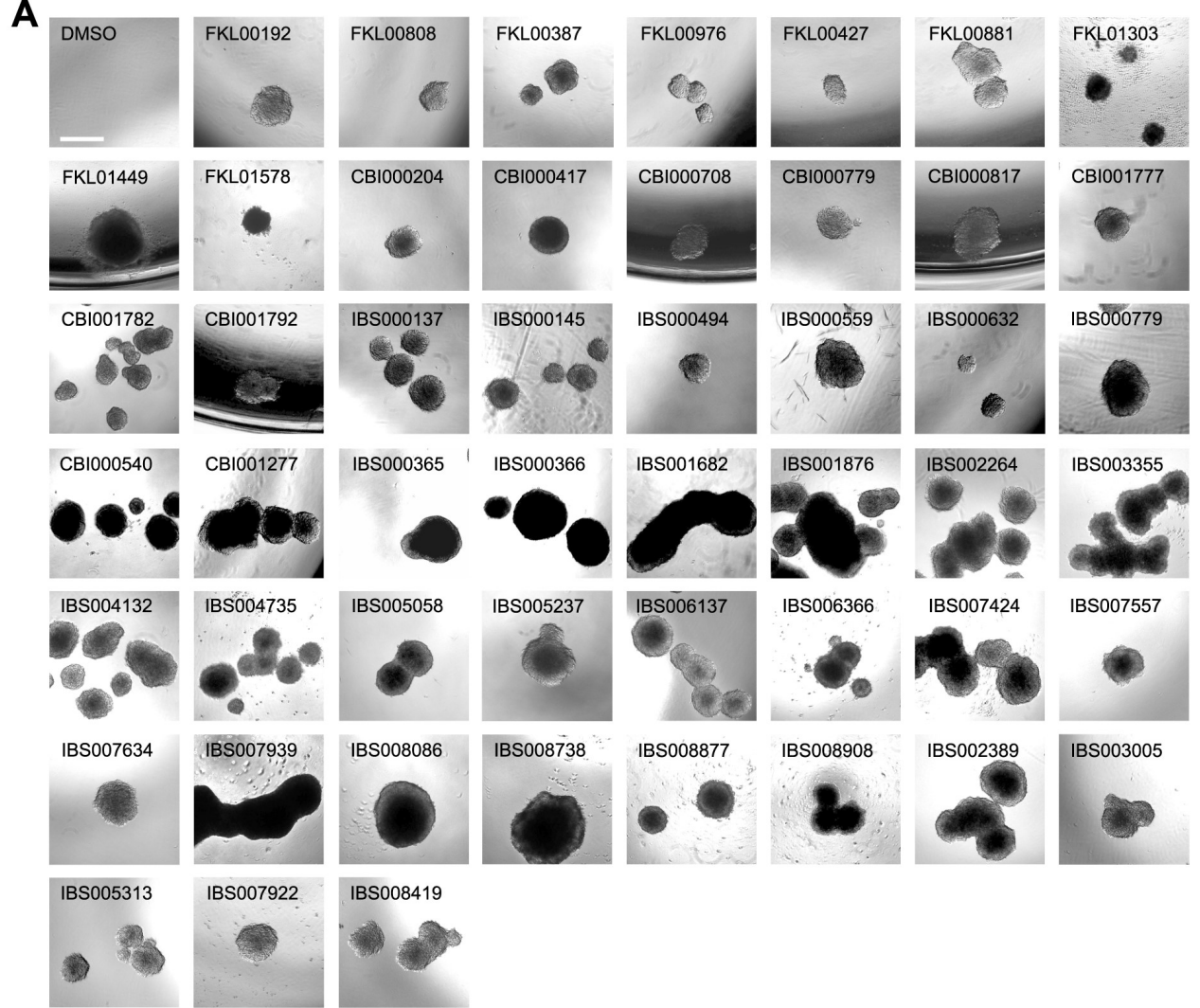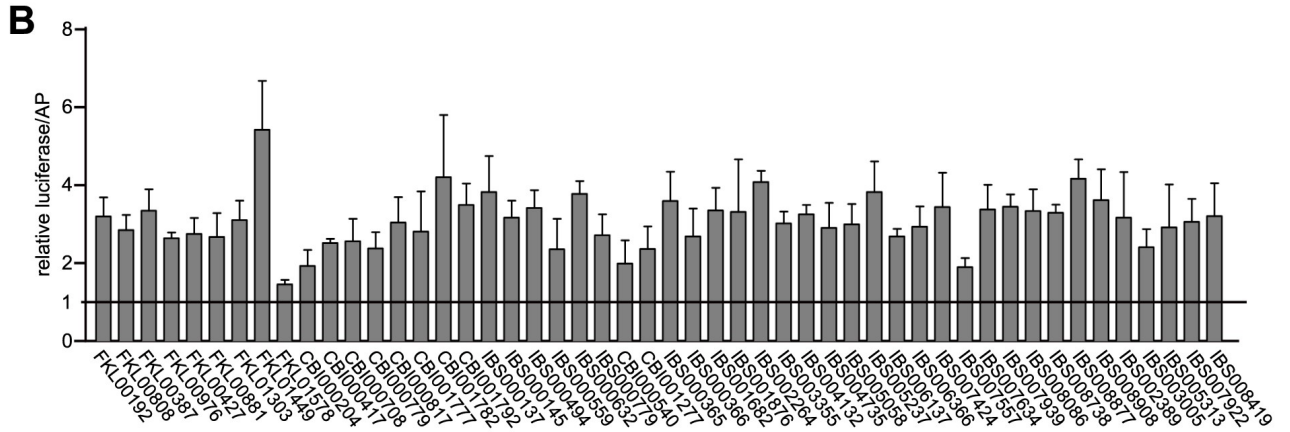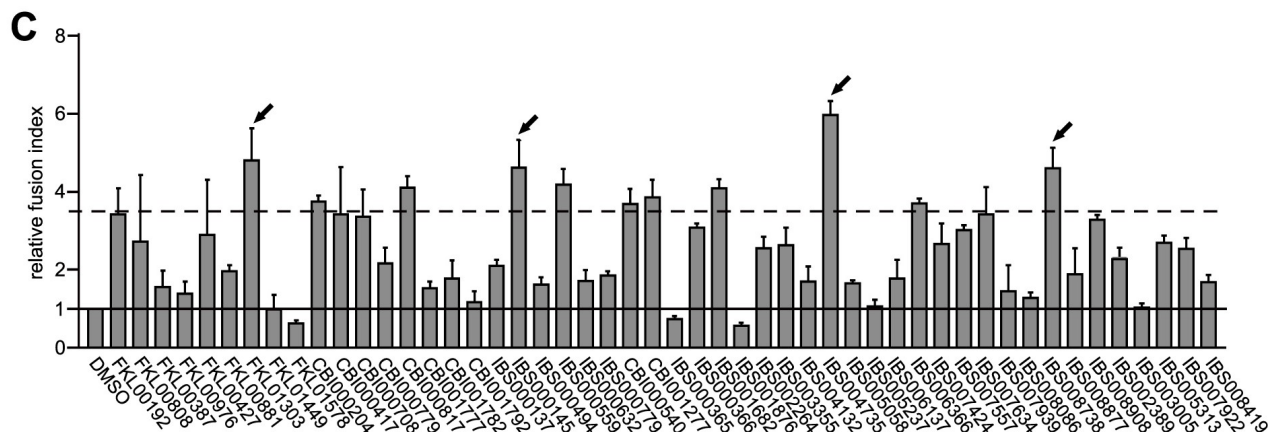

Supplementary Fig.1 Kodaka et al.

Supplement: S1 Fig — (PDF) [file pone.0231265.s001.pdf]

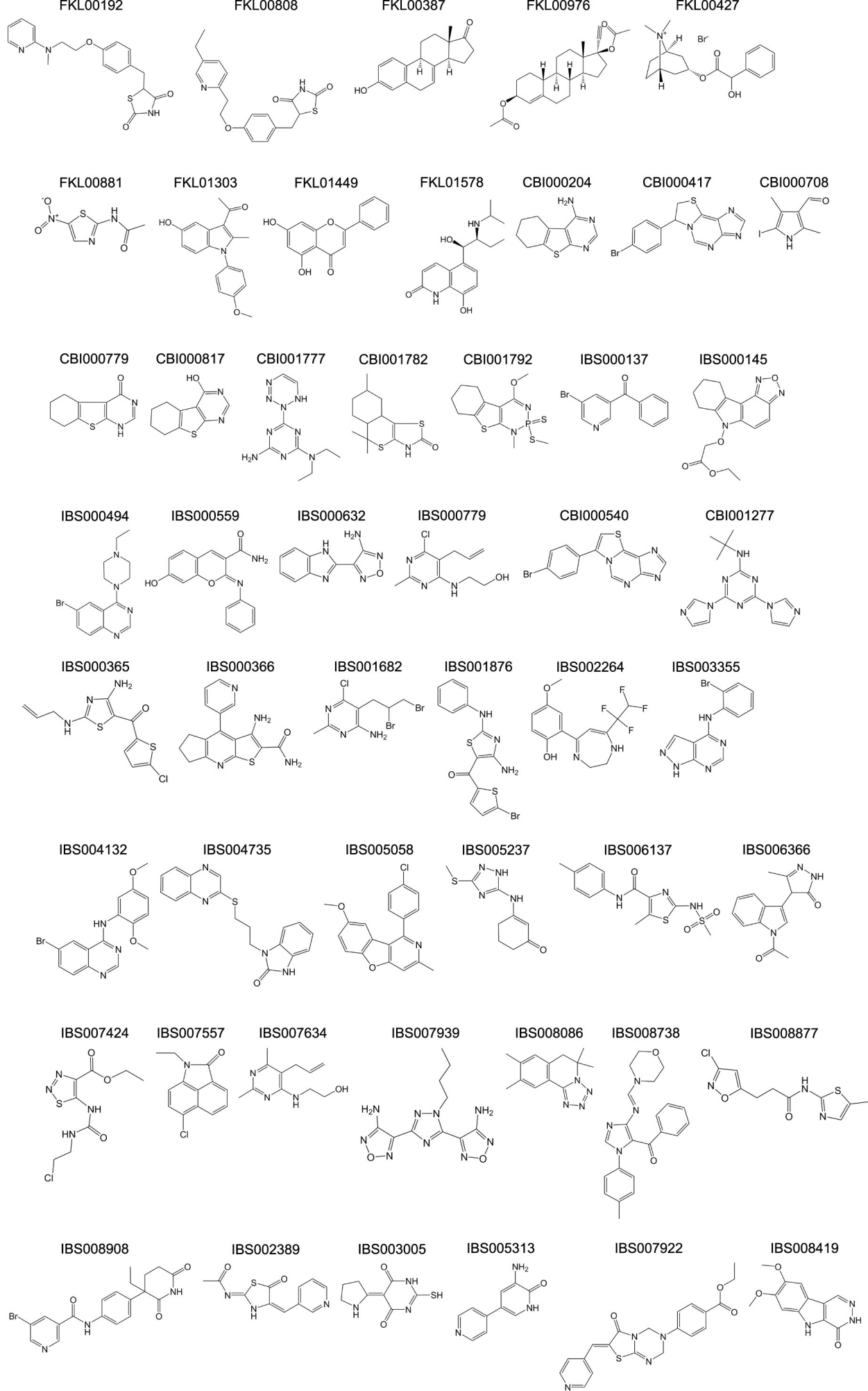

Supplementary Fig.2 Kodaka et al.

Supplement: S2 Fig — (PDF) [file pone.0231265.s002.pdf]

**A**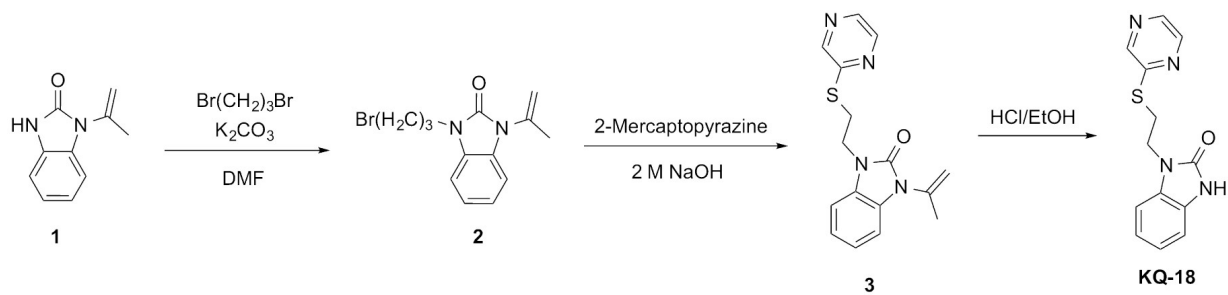**B**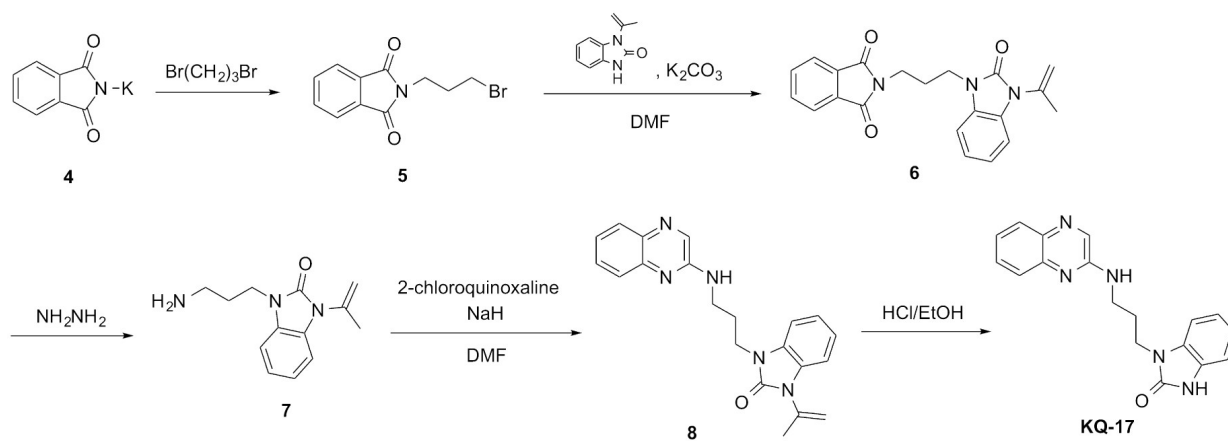

Supplement: S3 Fig — (PDF) [file pone.0231265.s003.pdf]

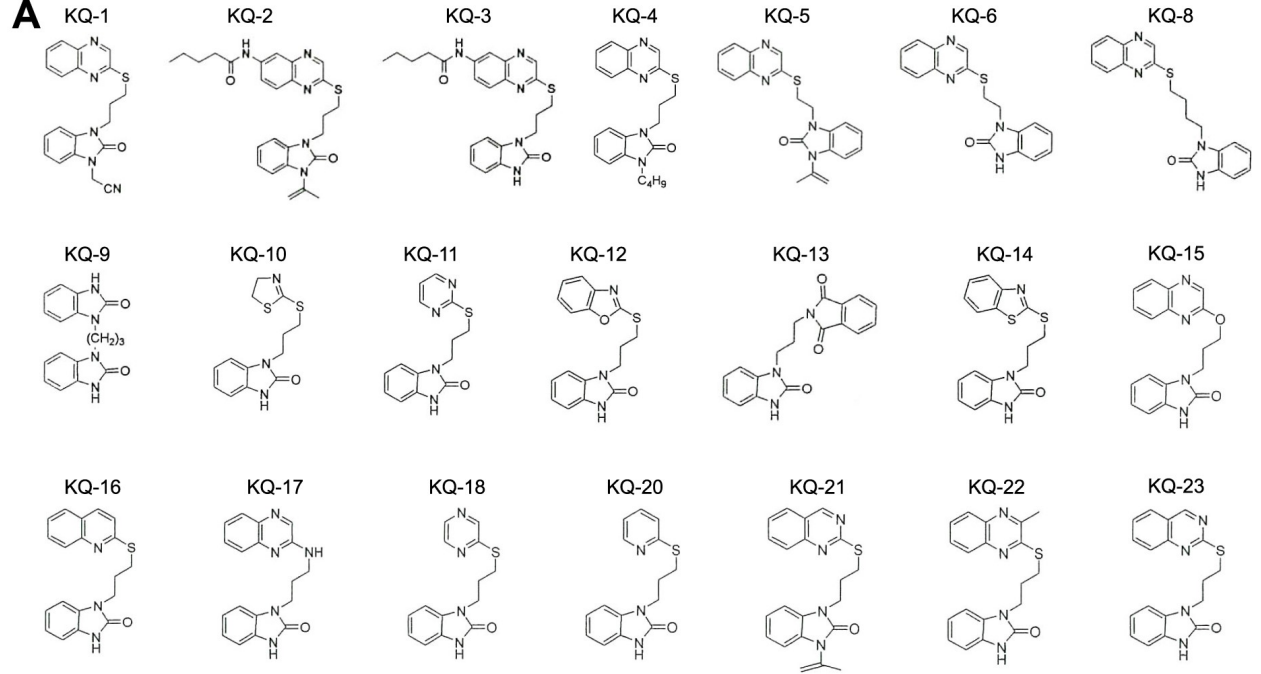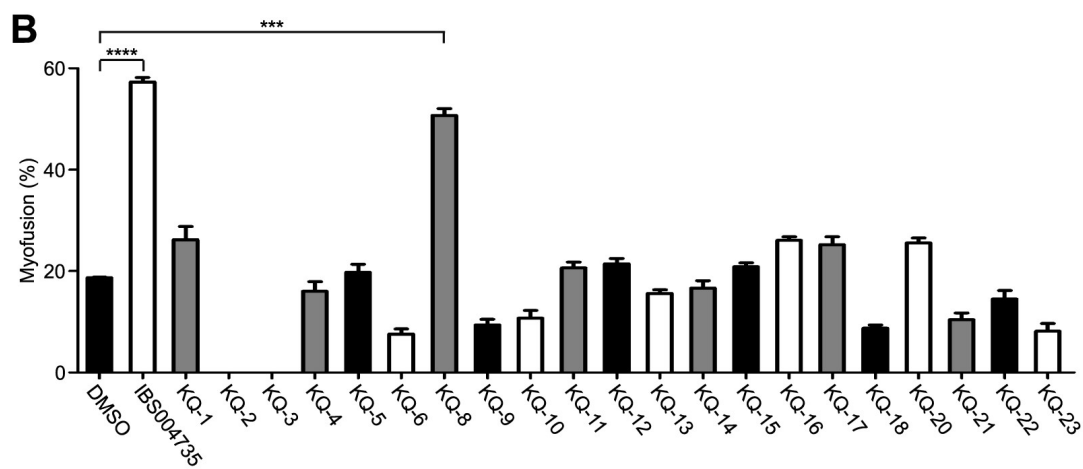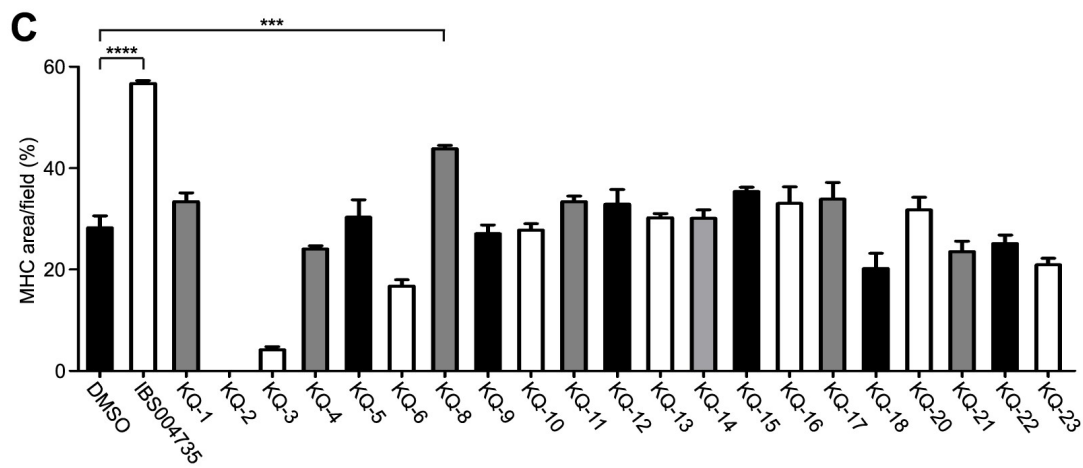

Supplement: S4 Fig — (PDF) [file pone.0231265.s004.pdf]

**A**

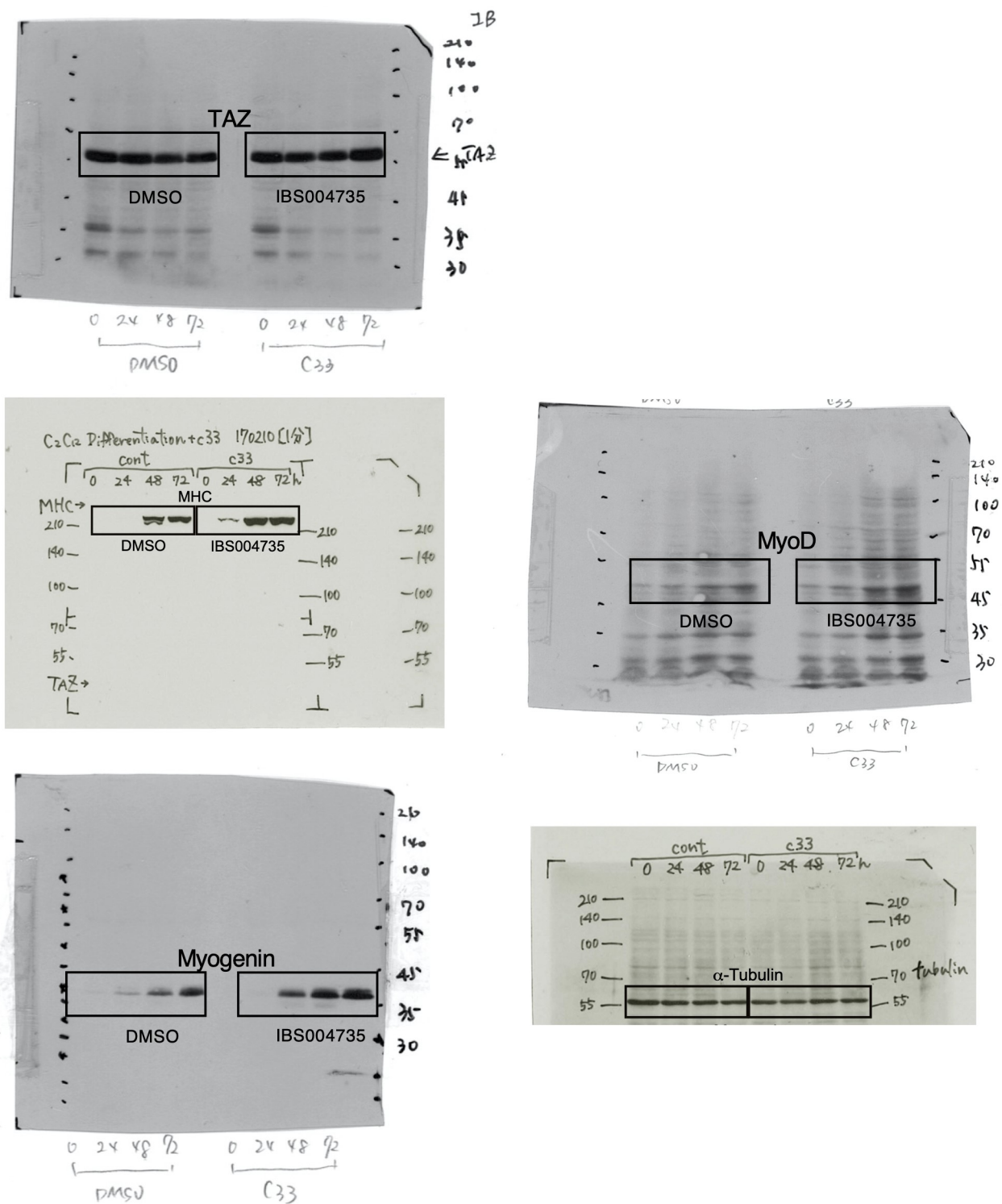

**B**

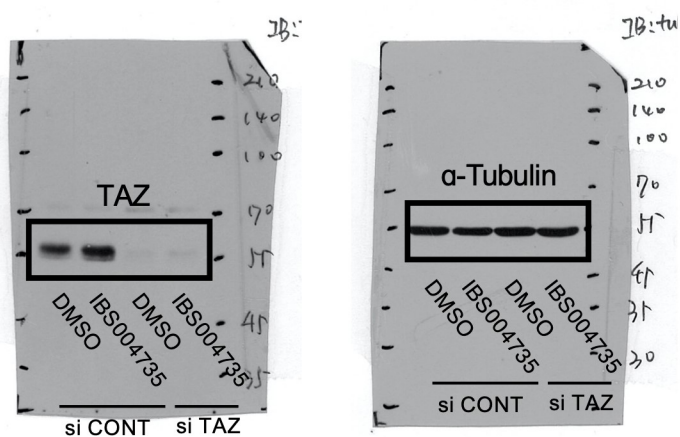

Supplementary Fig.5 Kodaka et al.

Supplement: S5 Fig — (PDF) [file pone.0231265.s005.pdf]

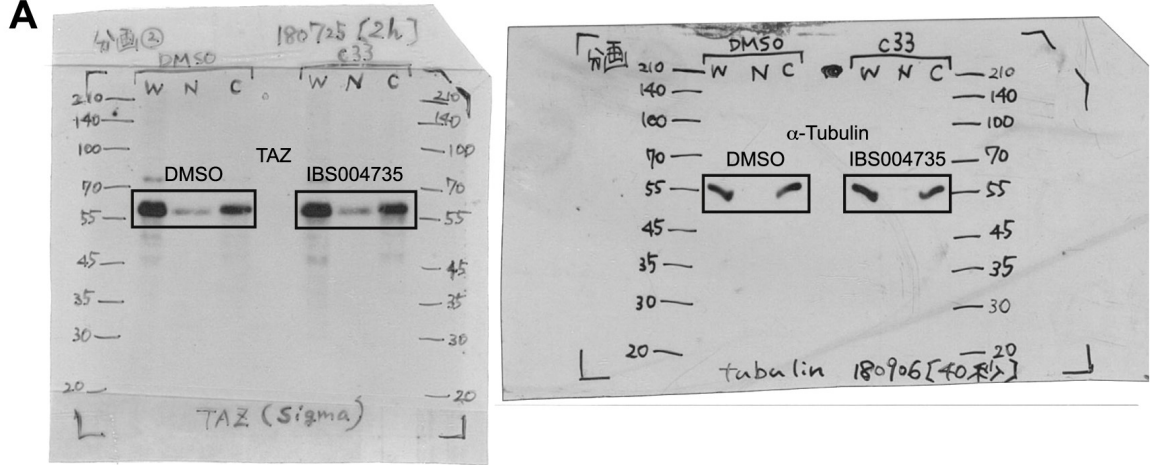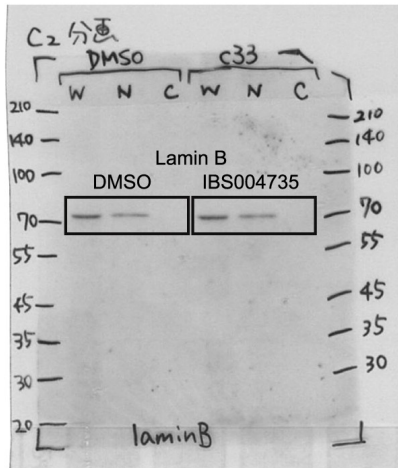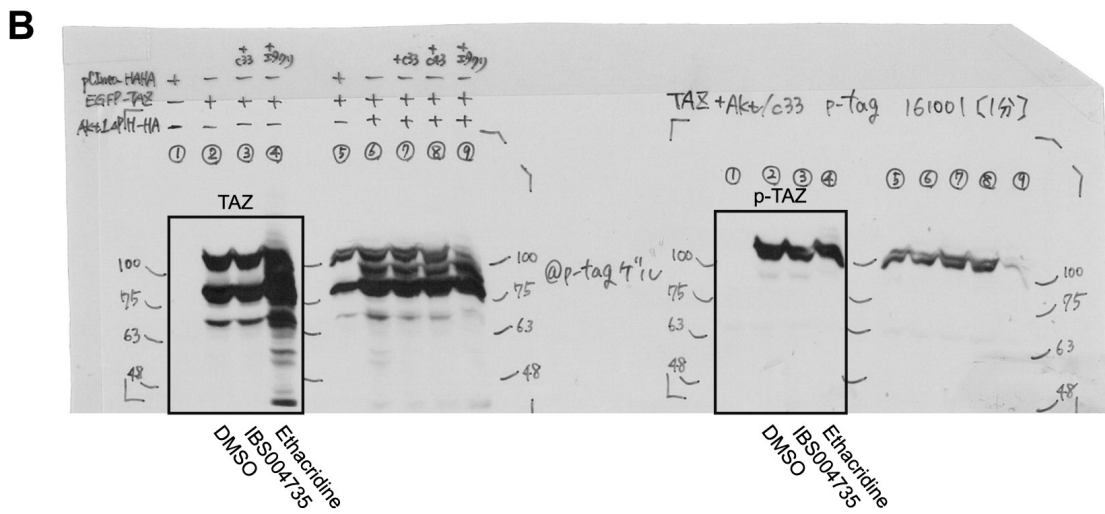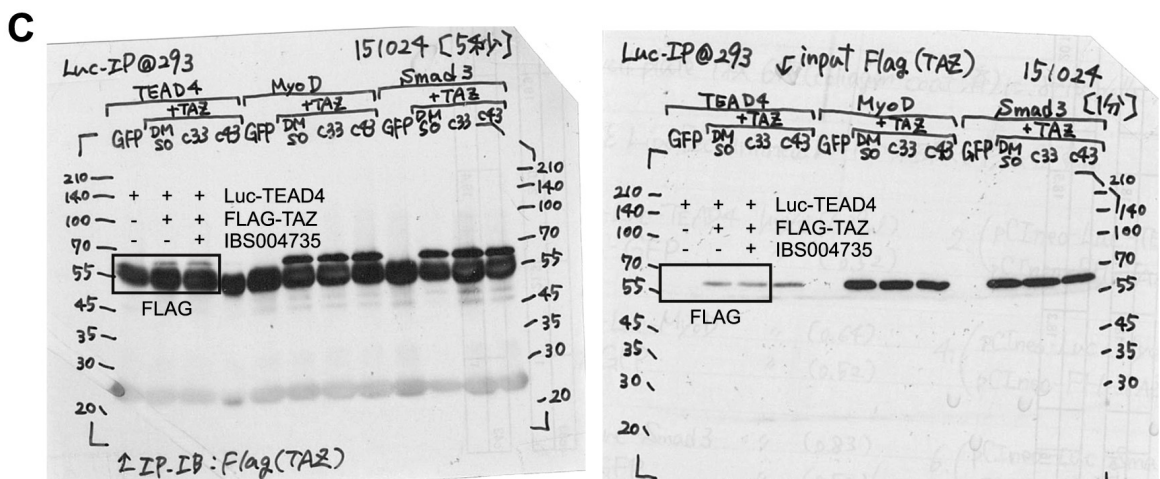

Supplementary Fig.6 Kodaka et al.

Supplement: S6 Fig — (PDF) [file pone.0231265.s006.pdf]

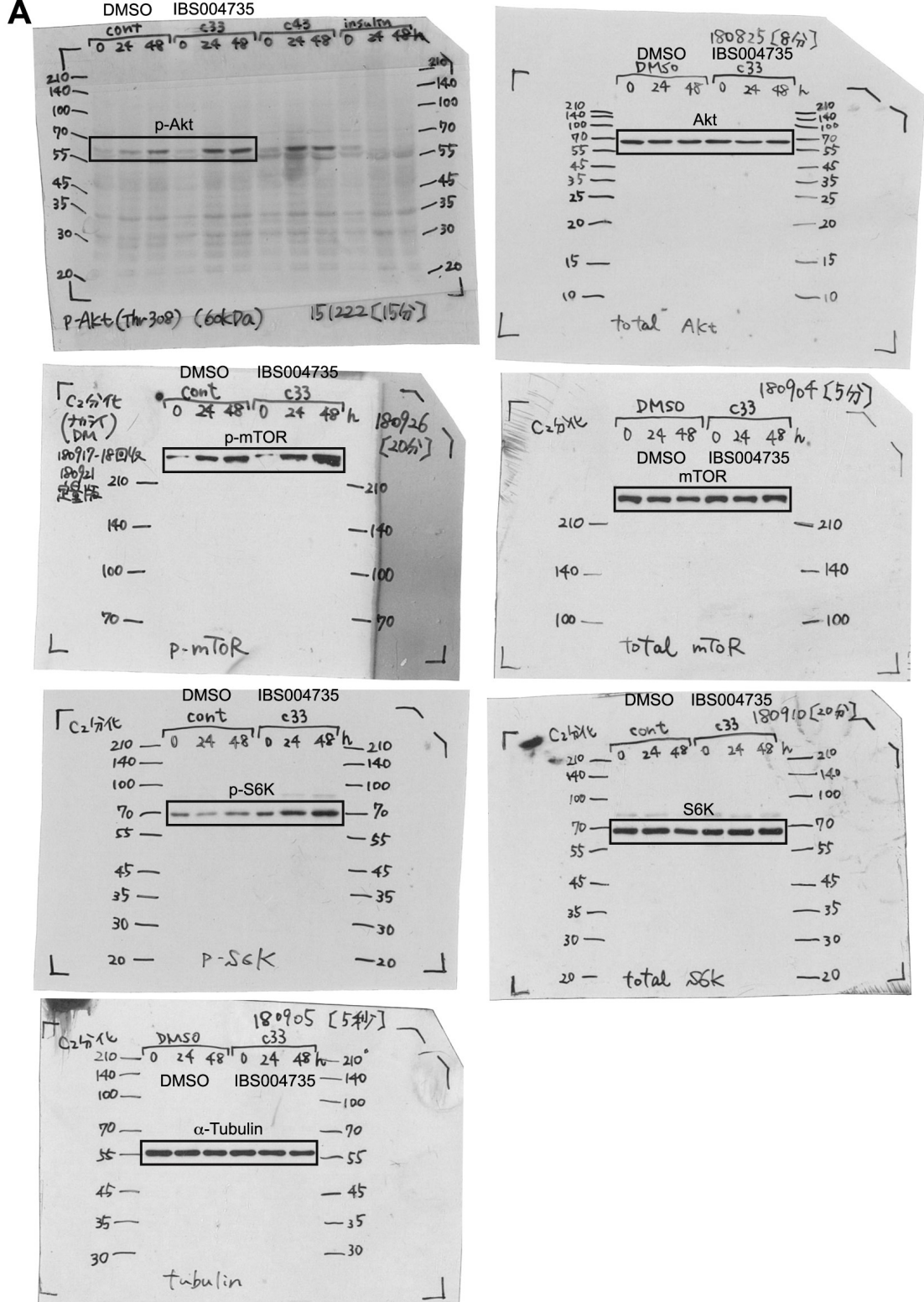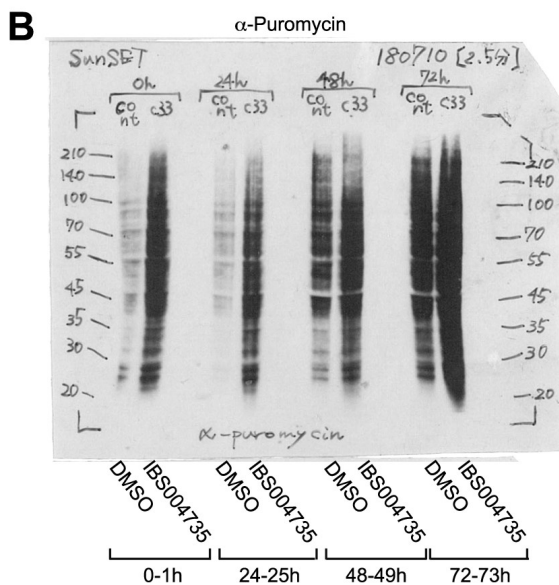

Supplementary Fig.7 Kodaka et al.

Supplement: S7 Fig — (PDF) [file pone.0231265.s007.pdf]

**A**

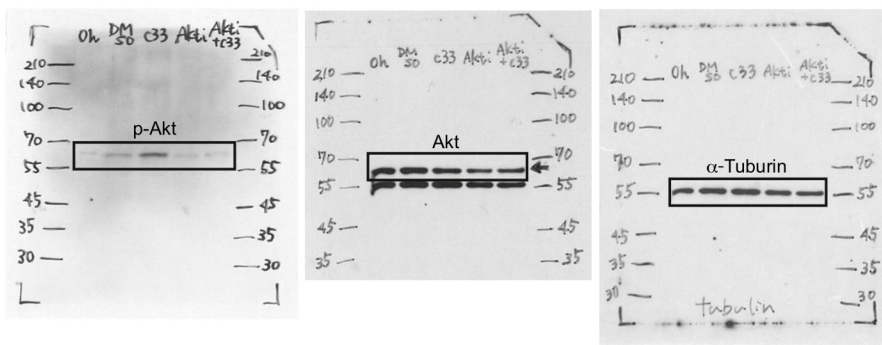

**B**

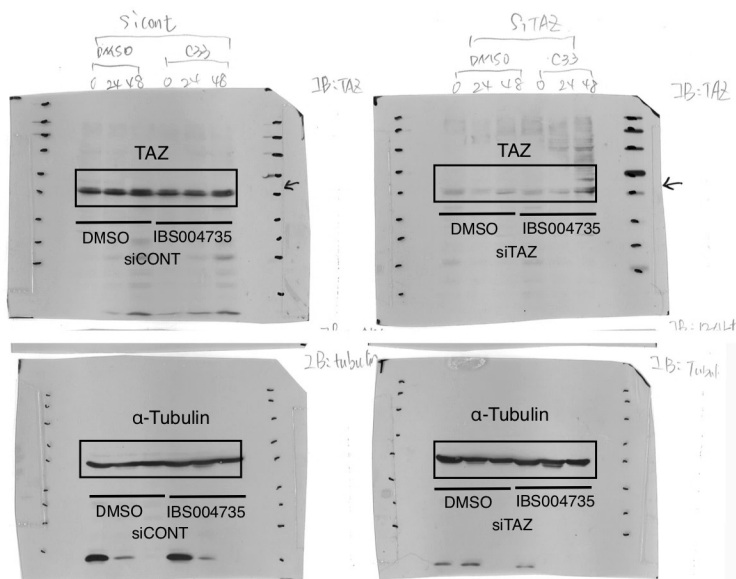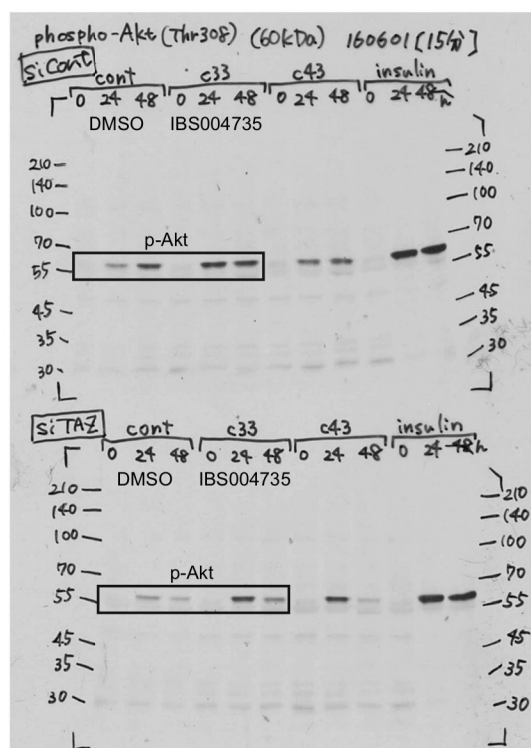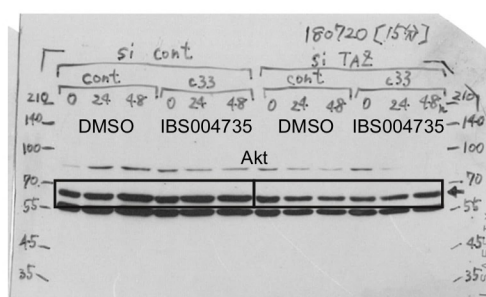

Supplementary Fig.S8 Kodaka et al.

Supplement: S8 Fig — (PDF) [file pone.0231265.s008.pdf]

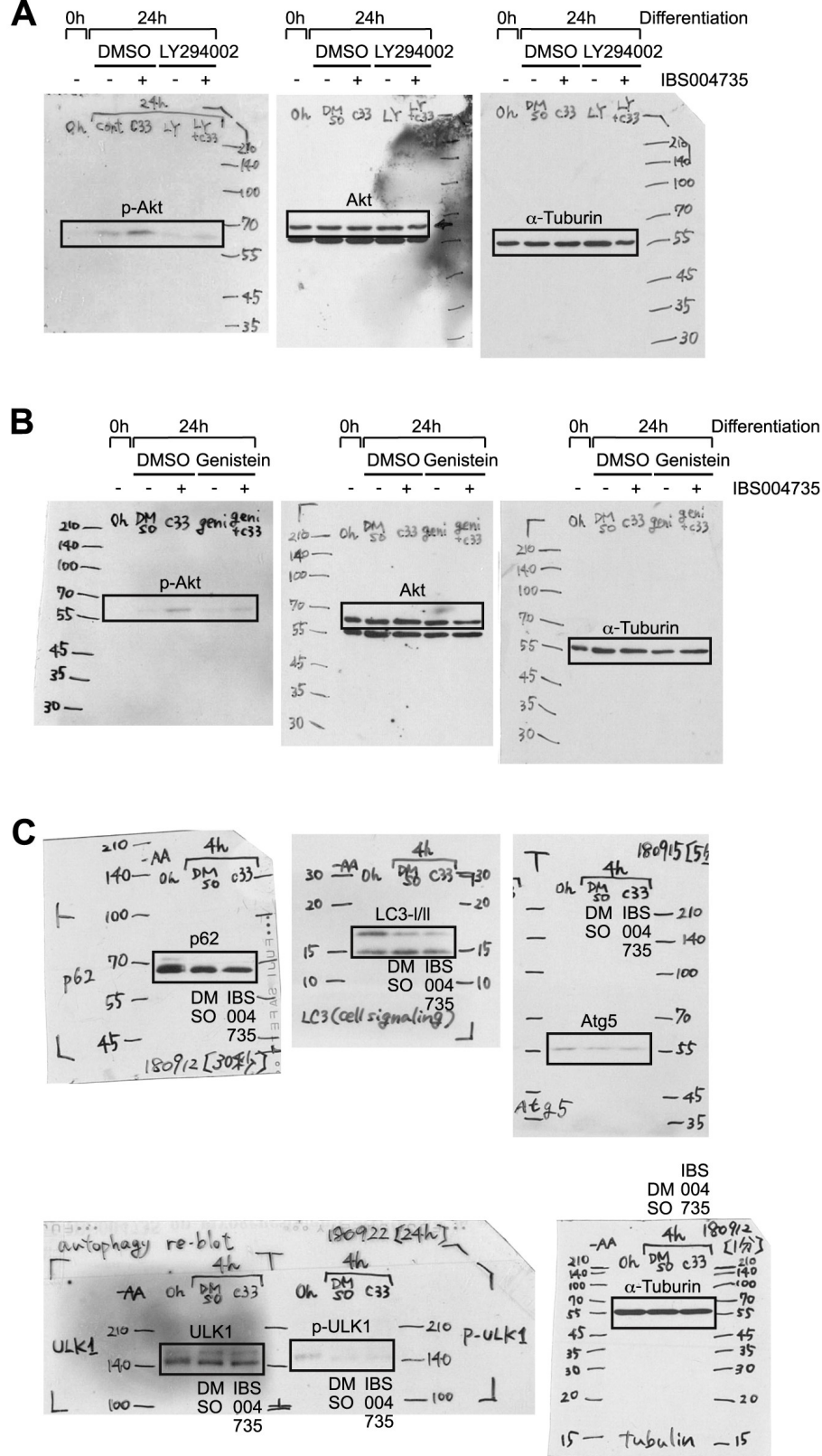

Supplementary Fig.9 Kodaka et al.

Supplement: S9 Fig — (PDF) [file pone.0231265.s009.pdf]

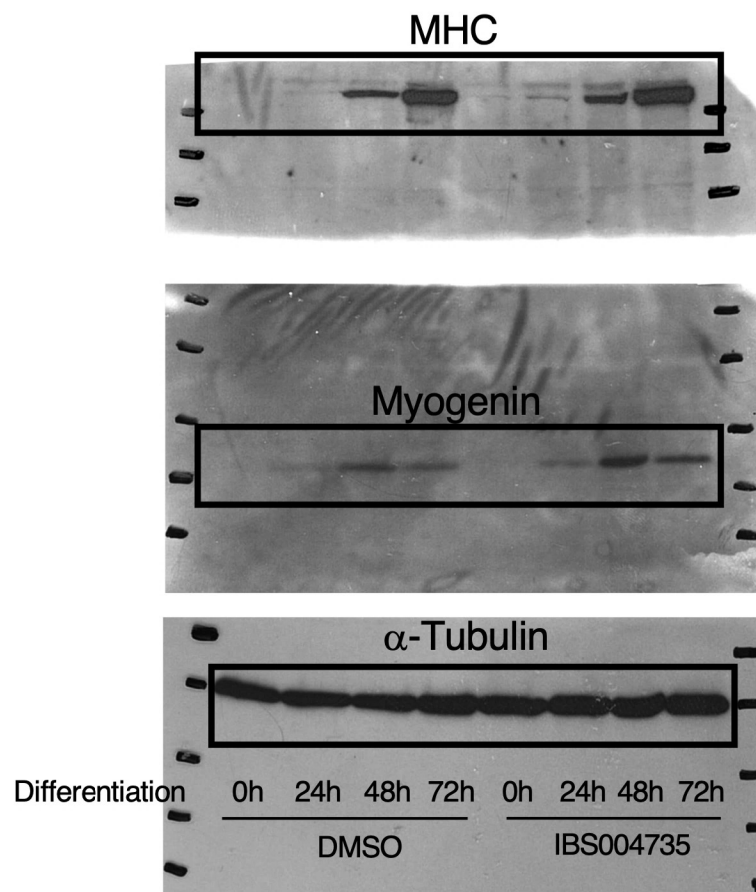

Supplementary Fig.10 Kodaka et al.

Supplement: S10 Fig — (PDF) [file pone.0231265.s010.pdf]
