# Supplementary material for: Characterization of a novel compound that promotes myogenesis via Akt and transcriptional co-activator with PDZ-binding motif (TAZ) in mouse C2C12 cells
Source: PLoS One. 2020 Apr 8;15(4):e0231265. doi: 10.1371/journal.pone.0231265 (PMC7141682; doi:10.1371/journal.pone.0231265)
Supplement: S1 Table — (DOCX) [file pone.0231265.s011.docx]

**Supplementary Table 1: The list of the antibodies**

| Name | Type | Application | Dilution | Company | Catalog number |
| --- | --- | --- | --- | --- | --- |
| TAZ | mouse monoclonal | WB, IF | 1:1000, 1:200 | BD Pharmingen | 560235 |
| MyoD | rabbit polyclonal | WB, ChIP | 1:1000, 1:100 | Santa Cruz | sc-760 |
| LaminB (M-20) | goat polyclonal | WB | 1:200 | Santa Cruz | sc-6217 |
| MHC | mouse monoclonal | WB, IF | 1:1000, 1:2000 | R&D Systems | MAB4470 |
| Laminin | rabbit polyclonal | IF | 1:500 | Sigma-Aldrich | L9393 |
| α-Tubulin | mouse monoclonal | WB | 1:1000 | Sigma-Aldrich | T9026 |
| FLAG M2 | mouse monoclonal | WB | 1:1000 | Sigma-Aldrich | F3165 |
| Puromycin, clone 12D10 | mouse monoclonal | WB | 1:1000 | Millipore | MABE343 |
| Pax3 | goat polyclonal | ChIP | 1:250 | Genway Biotech Inc. | GWB-3AE0A5 |
| TEAD4 | rabbit polyclonal | ChIP | 1:250 | Aviva | ARP38276-P050 |
| phospho-Akt (Thr308) | rabbit polyclonal | WB | 1:1000 | Cell signaling | #9275 |
| Akt | rabbit polyclonal | WB | 1:1000 | Cell signaling | #9272 |
| Phospho-mTOR (Ser2448) | rabbit polyclonal | WB | 1:1000 | Cell signaling | #5536 |
| mTOR (7C10) | rabbit monoclonal | WB | 1:1000 | Cell signaling | #2983 |
| Phospo-p70 S6 kinase (Thr389) | rabbit polyclonal | WB | 1:1000 | Cell signaling | #9205 |
| p70 S6 kinase | rabbit polyclonal | WB | 1:1000 | Cell signaling | #9202 |
| ULK1 (D8H5) | rabbit monoclonal | WB | 1:1000 | Cell signaling | #97094 |
| Phospho-ULK1 (Ser555) (D1H4) | rabbit monoclonal | WB | 1:1000 | Cell signaling | #97094 |
| Atg5 (D5F5U) | rabbit monoclonal | WB | 1:1000 | Cell signaling | #12994 |
| SQSTM1/p62 | mouse monoclonal | WB | 1:500 | Abcam | ab56416 |
| Myogenin | mouse monoclonal | WB | 1:1000 | Abcam | ab1835 |
| LC3-Ⅰ/Ⅱ | rabbit polyclonal | WB | 1:500 | Cell signaling | #2775 |
| Phospho-TAZ | rabbit polyclonal | WB | 1:1000 | House-made |  |
| GFP (B-2) | mouse monoclonal | WB | 1:1000 | Santa Cruz | sc-9996 |
|  |  |  |  |  |  |
